# Ginsenoside Rb1 can ameliorate the key inflammatory cytokines TNF-α and IL-6 in a cancer cachexia mouse model

**DOI:** 10.1186/s12906-019-2797-9

**Published:** 2020-01-15

**Authors:** Shuai Lu, Yubo Zhang, Huajun Li, Jing Zhang, Yingqian Ci, Mei Han

**Affiliations:** Key Laboratory of Radiopharmaceuticals, Ministry of Education, College of Chemistry, Beijing Normal University, 19 Xinjiekouwai Street, Haidian district, Beijing, China

**Keywords:** Cancer cachexia, Mouse model, Water extract of ginseng, Ginseng extract, Ginsenoside Rb1, TNF-α, IL-6

## Abstract

**Background:**

Cancer cachexia is a severe condition that leads to the death of advanced cancer patients, and approximately 50~80% of cancer patients have cancer cachexia. Ginseng extract has been reported to have substantial anticancer and immune-enhancing effects; however, no study has reported the use of ginseng alone to treat cancer cachexia. Our study’s purpose was to investigate the therapeutic effects of ginseng-related monomers or mixtures on a cancer cachexia mouse model.

**Methods:**

We selected BALB/c mice and injected the mice subcutaneously with C26 colon cancer cells to construct a cancer cachexia experimental animal model. The water extract of ginseng (WEG), two types of ginseng extracts (ginsenosides at doses of 5 mg/kg (GE5) and 50 mg/kg (GE50)) and ginsenoside Rb1 (Rb1) were used to treat cancer cachexia mice. Enzyme-linked immunosorbent assays (ELISAs) were used to analyze the inhibitory effects on two key inflammatory cytokines, tumor necrosis factor-α (TNF-α) and interleukin-6 (IL-6).

**Results:**

Our experimental results show that GE5, GE50 and Rb1 significantly reduced the levels of TNF-α (*P* < 0.01) and IL-6 (*P* < 0.01), which are closely related to cancer cachexia; however, WEG, GE5, GE50 and Rb1 did not significantly improve the gastrocnemius muscle weight or the epididymal fat weight of mice with cancer cachexia.

**Conclusions:**

These results indicate that GE5, GE50 and Rb1 may be useful for reducing symptoms due to inflammation by reducing the TNF-α and IL-6 cytokine levels in cancer cachexia mice, thereby ameliorating the symptoms of cancer cachexia. Our results may be beneficial for future studies on the use of Chinese herbal medicines to treat cancer cachexia.

## Background

Cancer cachexia usually affects advanced cancer patients, and it is a serious syndrome. Approximately 50–80% of all cancer patients will eventually suffer from cancer cachexia, and almost one-fifth of cancer deaths are caused by cancer cachexia [1]. Cancer cachexia involves a range of coordinating symptoms, including body weight and skeletal muscle loss, white adipose tissue dysfunction and systemic inflammatory responses. It has already been defined as a multifactorial syndrome, and it is impossible to wholly reverse this syndrome with only nutritional support as a treatment [2]. There are many types of cytokines that are related to cancer cachexia, including interleukins, interferons and tumor necrosis factors. Specifically, TNF-α and IL-6 are two key cytokines, and they mainly cause proteolysis and energy expenditure through related pathways [3]. Currently, there is no approved medicine for treating cancer cachexia, so many people are highly invested in researching cancer cachexia. Treatment should focus on how to stop reducing food intake (through nutritional support), how to reduce inflammation-related metabolic changes (through anti-inflammatory medicine or nutrients) and how to prevent the reduction of body weight and skeletal muscle weight [4].

Taking herbs to treat cancer is widely practiced around the world, and the use of herbal remedies increases from 5.3% before a cancer diagnosis to 13.9% after a cancer diagnosis [5]. A review shows that approximately 31.4% (from 7 to 64%) of adult patients suffering from cancer cachexia have been treated by complementary and alternative medicines [6], which indicates that herbal medicines are becoming increasingly popular. In the Asia-Pacific region, many traditional prescriptions containing *Panax ginseng* are used to treat cancer cachexia. For example, Qing-Shu-Yi-Qi Tang can stimulate the antitumor immune function of experimental animal models with chemotherapy [7]. The Baoyuan Jiedu Decoction is able to improve the quality of life and prevent muscle atrophy in cancer cachexia mice [8]. Rikkunshito can ameliorate cancer cachexia symptoms in a cancer cachexia rat model [9, 10]. Sipjeondaebo-tang can inhibit the production of IL-6 in a CT-26 cancer cachexia model [11]. In addition, the Buzhong Yiqi Decoction has the potential to treat cancer cachexia [12]. All in all, ginseng is one of the main ingredients in these prescriptions that may be effective against cancer cachexia. It is also believed that ginseng has the potential to treat cancer cachexia.

*Panax ginseng* is listed in the Chinese Pharmacopoeia, and it has a variety of pharmacological activities, including immunomodulatory abilities, which have significant effects on restoring physical strength, especially weakness and fatigue. Ginseng is one of the most widely used herbs, and it can enhance circulation, increase the blood supply, and can help patients recover from weakness caused by the disease [13]. In recent years, modern pharmacological studies have demonstrated that ginsenosides (the primary active components of *Panax ginseng*, including Rg3, Rh2, Re, Rb1, and Rg1) have strong immune regulation, anti-inflammatory, antioxidant and anticancer properties [14]. Ginsenoside Rb1 can reduce the release of TNF-α and IL-6 in a rat model of bone pain caused by cancer [15] and it can inhibit the expression of TNF-α and IL-6 induced by intestinal ischemia/reperfusion injury in rats [16]. The pro-inflammatory cytokines TNF-α and IL-6 in adipose tissue and liver in obese mice fed a high-fat diet [17] and the levels of TNF-α and IL-6 in postoperative ileus rats can be reduced by ginsenoside Rb1 [18]. In addition, epidemiological studies have shown that patients taking ginseng have a 50% lower risk of cancer recurrence compared with the risk of patients who do not take ginseng [19]. Studies have shown that ginseng partially regulates the host defense mechanisms. Experimental animal models used in in vivo studies have shown that ginseng extract enhances the activities of natural killer cells, phagocytosis and interferon production, which may be a potential mechanism for treating weakness and fatigue [20]. Currently, no studies have been reported on the evaluation of cancer cachexia by after treatment with a ginseng mixture or ginsenosides alone.

In China, the traditional Chinese medicine *Panax ginseng* is often used in the form of a water extract, and ginsenosides are the main active ingredients of ginseng extract. In our study, we evaluated the effect of *Panax ginseng*, including the water extract of ginseng, ginseng extract (a mixture containing a variety of ginsenosides) and ginsenoside Rb1, on cancer cachexia. We chose the C26-induced cancer cachexia model because it is a classic cancer cachexia mouse model [21]. The investigation was focused on the changes in the levels of TNF-α and IL-6 in mouse serum and changes in the body weights and gastrocnemius muscle weights of cancer cachexia mice to investigate whether ginseng is helpful for treating cancer cachexia. We demonstrated that ginseng can adjust the increased TNF-α and IL-6 cytokine levels to be appropriately decreased and we found that ginsenoside Rb1 plays a key role in the effectiveness of ginsenoside. This provides new ideas for therapies for cancer cachexia with ginseng-related medicine.

## Methods

### Cell culture

C26 colon adenocarcinoma cells were provided by the College of Life Sciences at Beijing Normal University. We used Dulbecco’s modified Eagle’s medium (DMEM, Gibco, USA) supplemented with 10% fetal bovine serum (FBS, Yuanhengjinma, Beijing, China) and 1% streptomycin-penicillin (Invitrogen, USA) to culture C26 colon adenocarcinoma cells. The temperature of the cell incubator was 37 °C, and the concentration of carbon dioxide was 5%.

### Ginseng extracts preparation

Dry ginseng pieces were purchased from Beijing Tongrentang Co., Ltd. (Beijing, China). A 250 mL round-bottom flask was used to load 13.5 g of ground ginseng pieces and 108 mL of distilled water (eightfold the amount of ginseng) was added; the mixture underwent a heating and condensing reflux for 1 h. The extract solution was cooled and filtered, and the above process was repeated with the residual liquid. The extract solution was combined, concentrated by heating, and the water extract of ginseng (WEG) was obtained at a final volume of 100 mL. The quality control was executed through the ginseng-related guide in the Pharmacopoeia of the People’s Republic of China (2015) (Additional file 1). We purchased ginseng extract dry powder (HPLC purity > 65%, Rg1: 3.49%, Re: 8.6%, Rf: 1.46%, Rb1: 14%, Rc: 16.4%, Rb2: 11.69%, Rd.: 9.5%; batch number: FY170314-B12) (Additional file 2) from Nanjing Feiyue Biological Technology Co., Ltd. (Nanjing, China) and dissolved it in phosphate-buffered saline (PBS) to form different ginseng extract solutions before use. Based on the ginsenoside content, ginseng extracts were prepared as follows: ginsenosides at doses of 5 mg/kg (GE5) and ginsenosides at doses of 50 mg/kg (GE50). Ginsenoside Rb1 was purchased from Shanghai Harling Biotechnology Co., Ltd. (HPLC purity > 98%, Shanghai, China). Ginsenoside Rb1 (Rb1) was formulated in PBS at a dose of 10.72 mg/kg to match the concentration of ginsenoside Rb1 in GE50.

### Experimental animals

Male BALB/c mice (the mice were aged 4–6 weeks, and the body weight was 18–22 g) were provided by Beijing Vital River Laboratory Animal Technology Co., Ltd. and they were caged in groups of six in a controlled temperature and humidity room for 12 h: 12 h light/dark cycle with free access to water and standard rodent food. All experimental animal-related experiments were given official approval by the Ethics Committee of Beijing Normal University (approved case number: BNU/EC/01/2011, date: 07/07/2011). All experimental procedures were performed in accordance with the 1996 Guide for the Care and Use of Laboratory Animal (NIH Publications No. 80–23).

### Experimental procedures

In the first experiment, after 18 BALB/c mice (the mice were aged 4–6 weeks, and the body weight was 18–22 g) were acclimated for a week, the experimental mice were separated in a random manner into the following 3 groups: the control group (normal mice administered with PBS, *n* = 6), model group (tumor-bearing mice administered with PBS, *n* = 6) and WEG group (tumor-bearing mice treated with WEG, *n* = 6). In the second experiment, 54 BALB/c mice (the mice were aged 4–6 weeks, and the body weight was 18–22 g) were separated in a random manner into 5 groups: the control group (normal mice administered with PBS, *n* = 11), model group (tumor-bearing mice administered with PBS, *n* = 11), GE5 group (tumor-bearing mice treated with GE5, *n* = 10), GE50 group (tumor-bearing mice treated with GE50, *n* = 11) and Rb1 group (tumor-bearing mice treated with Rb1, *n* = 11). On day 0, all mice from the model groups and the treatment groups were implanted with 100 μL of a cell suspension containing 1 × 10^6^ C26 cells (cell suspension configured by PBS) by subcutaneous injection in the right limb to build the cancer cachexia model, and 100 μL PBS was implanted by the same injection method into each mouse in the control groups. The whole inoculation process was completed within one hour. After the inoculation, all mice were housed in a day and night cycle room (lighting time: 08:30–20:30) and were free to eat. The body weight of the mice was weighed every two days. The activity of the mice and the tumor growth were observed. The tumor volume was measured using a digital Vernier caliper. The mice in the control and model groups received daily PBS orally from the first day to the twenty-third day. The mice in the other treatment groups received each of the corresponding types and concentrations of drugs orally. From the seventh day, the long and short diameters of the tumor were measured with a digital Vernier caliper every two days. Finally, the tumor volume was calculated according to the formula: tumor volume (V) = 1/2 a × b^2^, where a and b represent the tumor’s long diameter (mm) and short diameter (mm), respectively. The tumor weight was calculated by M = F × V, and F was calculated by comparing the tumor volume and the tumor weight on the last day. The tumor-free body weight was calculated by the body weight minus the tumor weight. On day 23, approximately 0.5 mL blood samples were collected and were allowed to settle for 30 min. The blood samples were centrifuged at 3000 rpm in a centrifuge for 15 min to obtain the serum. After the mice were sacrificed by cervical dislocation, the underlying tissues and organs were immediately removed and weighed, including the tumors, livers, hearts, spleens, lungs, kidneys, gastrocnemius muscles and epididymal fat.

### Detection of inflammatory cytokines

The inflammatory cytokines of TNF-α and IL-6 in the mouse serum were tested by using enzyme-linked immunosorbent assay (ELISA) kits (mice TNF-α kit and mice IL-6 kit; Mlbio, Shanghai, China) according to the manufacturer’s instructions after the sera were thawed for 30 min. The cytokine detection was performed by the company Shanghai Enzyme-linked Biotechnology Co., Ltd.

### Statistical analysis

The experimental results are expressed in the form of the mean and standard deviation. One-way ANOVA was performed using SPSS version 19.0 to analyze the data to compare the significant differences between the different groups. *P* values less than 0.05 were considered statistically significant. * *P* < 0.05, ** *P* < 0.01 represent the results of the comparison with the control group and # *P* < 0.05, ## *P* < 0.01 represent the results of the comparison with the model group.

## Results

### Effect of WEG, GE5, GE50 and Rb1 on the tumor-free weight

**Fig. 1:**
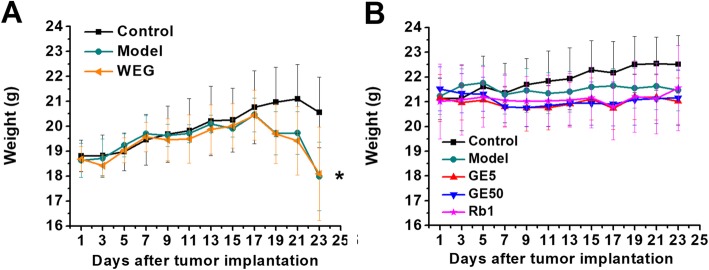
Effect of WEG, GE5, GE50 and Rb1 on the tumor-free body weight in C26 tumor-bearing mice. (**a**, **b**) The body weight of each mouse in all groups was measured every two days at the same time until the twenty-third day. (**a**) Control (normal mice administered with PBS, *n* = 6), Model (tumor-bearing mice administered with PBS, *n* = 6), WEG (tumor-bearing mice treated with WEG, *n* = 6); (**b**) Control (normal mice administered with PBS, *n* = 11), Model (tumor-bearing mice administered with PBS, *n* = 11), GE5 (tumor-bearing mice treated with GE5, *n* = 10), GE50 (tumor-bearing mice treated with GE50, *n* = 11), and Rb1 (tumor-bearing mice treated with Rb1, *n* = 11). Model group vs. Control group: * *P* < 0.05

The mice in the first experimental model group showed a significant decrease in their tumor-free body weights (Fig. 1a, *P* < 0.05) on the twenty-third day compared with that of control group. There were no significant changes in the tumor-free body weights of the WEG (Fig. 1a), GE5, GE50 and Rb1 groups (Fig. 1a) compared with those of each model group. All four different medicines (WEG, GE5, GE50 and Rb1) showed no ability to improve the tumor-free weight.

### Effect of WEG, GE5, GE50 and Rb1 on the weights of the epididymal fat and the gastrocnemius muscle

**Fig. 2:**
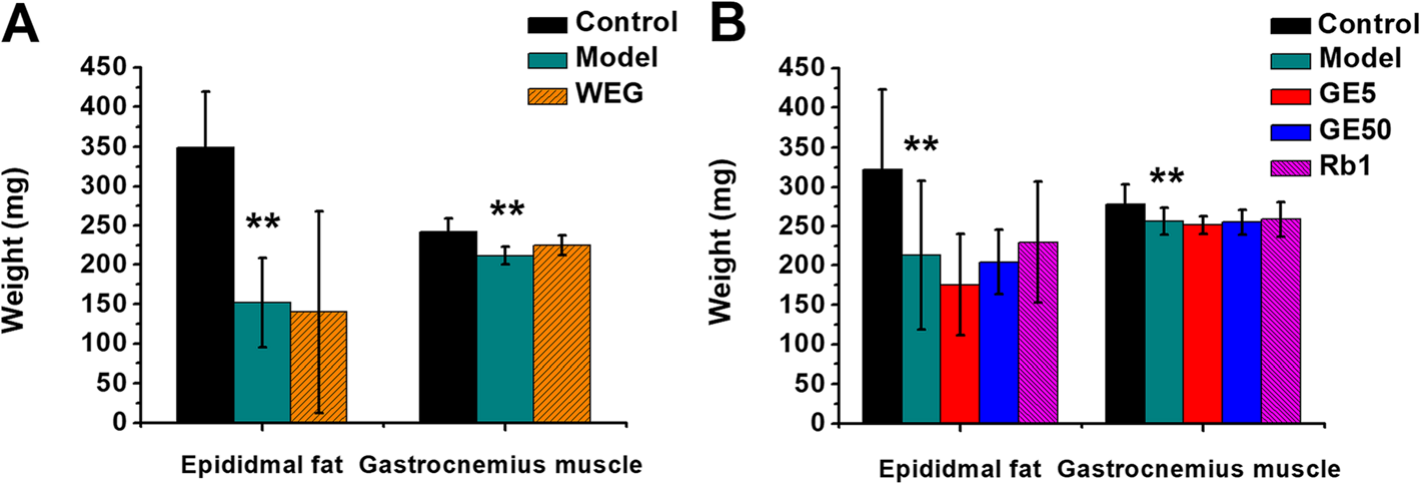
Effect of WEG, GE5, GE50 and Rb1 on the epididymal fat weight and the gastrocnemius muscle weight in C26 tumor-bearing mice. (**a**), (**b**) The weights of the epididymal fat and the gastrocnemius muscle were measured on the 23rd day at the time of sacrifice. Model group vs. Control group: ** *P* < 0.01

In the first experiment, the model group had significant decreases in the epididymal fat weight (Fig. 2a, *P* < 0.01) and in the gastrocnemius muscle weight (Fig. 2a, *P* < 0.01) compared with those of the control group. In the second experiment, the model group had a significant decrease in the weights of the epididymal fat (Fig. 2b, *P* < 0.01) and the gastrocnemius muscle (Fig. 2b, *P* < 0.01) compared with those of the control group. The two experimental results showed that the untreated tumor-bearing mice exhibited significant decreases in the weights of their epididymal fat and gastrocnemius muscles. All medicines (WEG, GE5, GE50 and Rb1) showed no improvement in the weights of the epididymal fat or the gastrocnemius muscle (Fig. 2) compared with those of each model group.

### Effect of WEG, GE5, GE50 and Rb1 on the cumulative food intake and tumor volume change

**Fig. 3:**
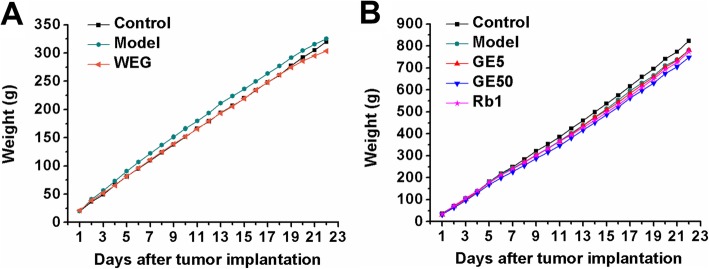
Effect of WEG, GE5, GE50 and Rb1 on the weight of the food intake of C26 tumor-bearing mice. (**a**), (**b**) Food intake was measured every day until the 22nd day. The results of the GE5 group were corrected by multiplying by 11/10

**Fig. 4:**
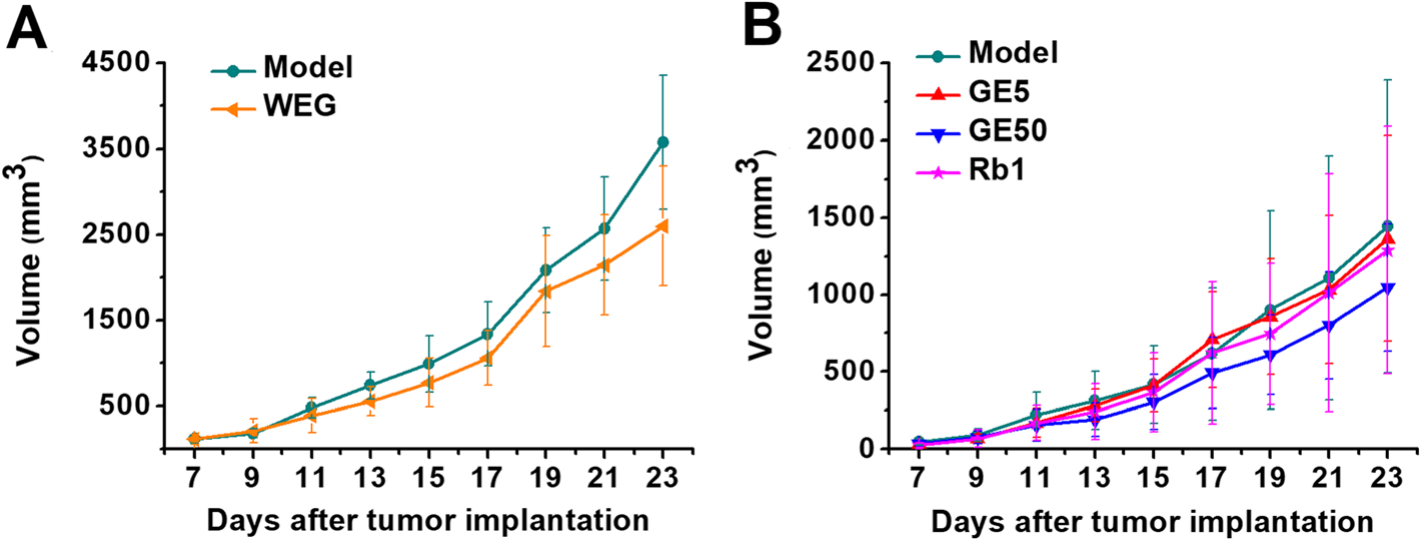
Effect of WEG, GE5, GE50 and Rb1 on tumor volume in C26 tumor-bearing mice. (**a**), (**b**) The volume was measured every two days from the 7th day to the last day

In the first and second experiments, there were no significant changes in the food intake (Fig. 3) or the tumor volume (Fig. 4) in the WEG, GE5, GE50 and Rb1 groups compared with those of each model group. All medicines (WEG, GE5, GE50 and Rb1) used in each treatment group showed no significant effects in reducing the tumor volume and increasing the food intake.

### Effect of WEG, GE5, GE50 and Rb1 on the weights of the livers, hearts, spleens, lungs and kidneys

**Fig. 5:**
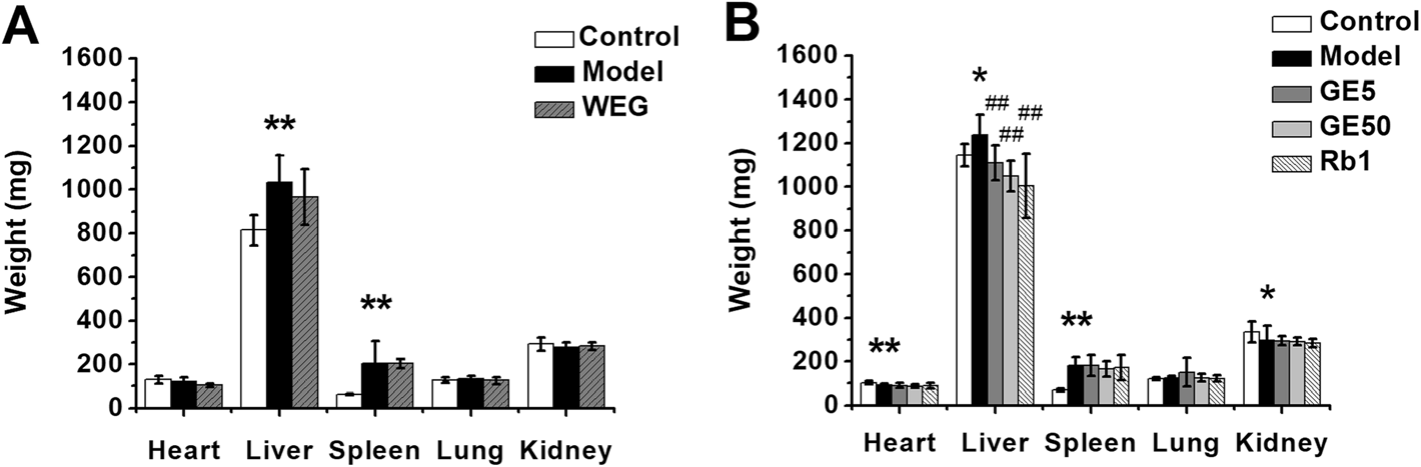
Effect of WEG, GE5, GE50 and Rb1 on the weights of the livers, hearts, spleens, lungs and kidneys in C26 tumor-bearing mice. (**a**), (**b**) The weight of the livers, hearts, spleens, lungs and kidneys were measured on the 23rd day at the time of sacrifice. Model group vs. Control group: * *P* < 0.05, ** *P* < 0.01; WEG group, GE5 group, GE50 group, Rb1 group vs. Model group: ## *P* < 0.01

In the first experiment, the liver weight was significantly increased (Fig. 5a, *P* < 0.01), and the spleen weight was significantly increased (Fig. 5a, *P* < 0.01) in the model group. The WEG group had no significant difference except for the decrease in the heart weight (Fig. 5a, *P* = 0.058) compared with that of the model group. In the second experiment, the liver weight was significantly increased in the model group (Fig. 5b, *P* < 0.05), and the spleen weight was significantly increased (Fig. 5b, *P* < 0.01). This situation is consistent with the results of the first experiment. Compared with that of the model group, GE5, GE50, and Rb1 caused significant decreases in the liver weight (Fig. 5b, *P* < 0.01). In addition, the heart weight had a significant reduction in the model group (Fig. 5b, *P* < 0.01), and the kidney weight had a significant reduction in the model group (Fig. 5b, *P* < 0.05) in the second experiment, but there was no significant difference in the heart weight or kidney weight in the first experiment. All groups showed no significant changes in the lung weight. Altogether, the livers and spleens of the tumor-bearing mice were larger than those of the normal mice, yet WEG cannot ameliorate the weight reduction of the liver and spleen; however, GE5, GE50, and Rb1 can help the liver weights of the cancer cachexia mice return to normal.

### WEG, GE5, GE50 and Rb1 can reduce the inflammatory cytokines TNF-α and IL-6 in the sera of cancer cachexia mice

**Fig. 6:**
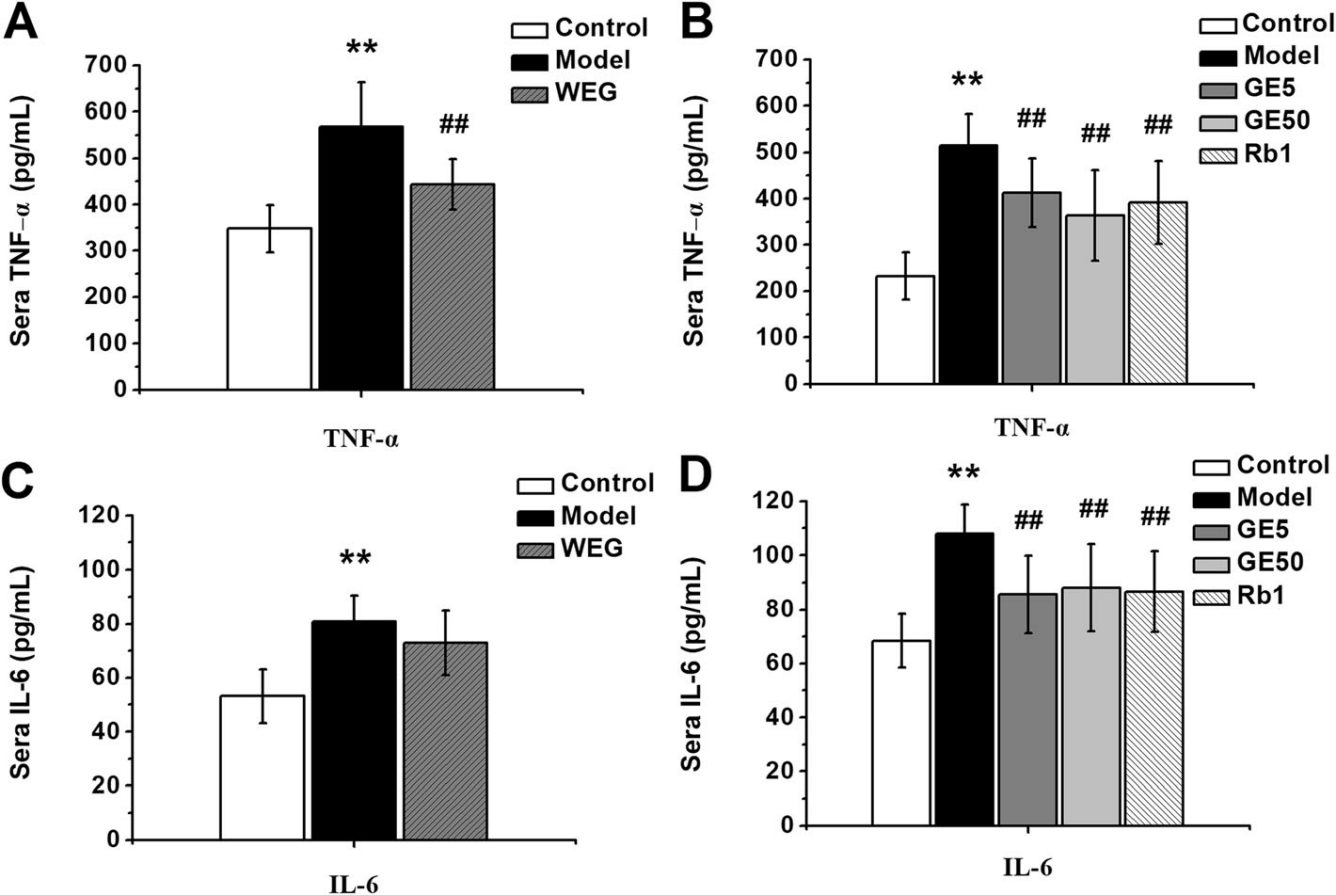
Effect of WEG, GE5, GE50 and Rb1 on the TNF-α and IL-6 levels in C26 cancer cachexia mouse sera. (**a**), (**c**) The TNF-α and IL-6 levels from the WEG-treated mouse sera were measured after the sera were collected in the first experiment (*n* = 6); (**b**), (**d**) The TNF-α and IL-6 levels from the GE5-, GE50- and Rb1-treated mouse sera were measured after the sera were collected in the second experiment (*n* = 10). Model group vs. Control group: ** *P* < 0.01; WEG group, GE5 group, GE50 group, Rb1 group vs. Model group: ## *P* < 0.01

In the first experiment, the TNF-α cytokine levels in the model group mouse sera were significantly increased (Fig. 6a, *P* < 0.01) compared with those of the control group. In the second experiment, the TNF-α cytokine levels in the model group mouse sera were similarly increased (Fig. 6b, *P* < 0.01). The TNF-α cytokine levels in the WEG group were significantly decreased (Fig. 6a, *P* < 0.01) compared with those of the model group. The three experimental groups (GE5, GE50 and Rb1) significantly reduced the TNF-α cytokine contents in the mouse sera (Fig. 6b, *P* < 0.01) compared with those in the model group, and the effects are better than those of WEG. IL-6 cytokines in the mouse sera were significantly increased in the model group (Fig. 6c and d, *P* < 0.01) compared with those of the control groups of the first and second experiments. WEG reduced the IL-6 cytokines in the mouse serum compared with those in the model group, but there was no significant difference in the IL-6 cytokine content (Fig. 6c, *P* > 0.05). GE5 and GE50 significantly reduced the IL-6 cytokine levels in the mouse sera (Fig. 6d, *P* < 0.01), and Rb1 also decreased the IL-6 cytokine levels in the mouse sera (Fig. 6d, *P* < 0.01) compared with those in the model group.

## Discussion

Cancer cachexia is a serious symptom of advanced cancer, and many people die because of cachexia [1]. There are still many problems in treating cancer cachexia, and there are no approved drugs. Therefore, many people try to use Chinese medicine to improve cancer cachexia [7–12]. In our study, we used *Panax ginseng* to try to improve the status of cancer cachexia mice. We found that GE5, GE50 and Rb1 can reduce the inflammatory cytokines TNF-α and IL-6 in cancer cachexia mice. This is a good reference for future studies to treat cancer cachexia by using Chinese herbal medicine, including *Panax ginseng*.

Cancer cachexia is a multiorgan syndrome, so the adipose tissue, liver, heart and other tissues/organs are directly involved in the cancer cachexia process [22]. To study the effects on the nonmuscle tissues and organs due to cancer cachexia, we examined the weight of the epididymal fat, livers, hearts, spleens, lungs and kidneys. By analyzing the results, we found that the weights of the livers and spleens of cachexia mice were significantly increased (*P* < 0.05) (Fig. 5a and b). The livers and spleens of mice with cancer cachexia are enlarged due to the influence of tumor factors. GE5, GE50 and Rb1 all reduced the liver weights of mice with cachexia (Fig. 5b, *P* < 0.01). The recovery of the organ weight also showed that the use of GE5, GE50 and Rb1 can help cancer cachexia mice to restore their physical condition to a certain extent, thereby alleviating the cancer cachexia syndrome.

Studies have suggested that cachexia is rarely attributable to only one cytokine but is attributable to a series of cytokines and to other cachexia-related factors that work synergistically [23]. TNF-α and IL-6 are two indicators of cancer cachexia, and the levels of these two cytokines are usually tested in many studies to determine the therapeutic effect [24, 25]. TNF-α is one of the inflammatory mediators associated with the development of carcinogenesis [26], and it plays a key role in the mechanisms of the loss of skeletal muscle, systemic inflammation, and the loss of adipose tissue in cancer cachexia [27]. WEG can reduce the level of TNF-α (Fig. 6a, *P* < 0.01). GE5, GE50 and Rb1 significantly reduced the levels of TNF-α (Fig. 6b, *P* < 0.01). IL-6 is considered to be a master regulator of the acute phase response in patients with cachexia and can raise tumor growth in the cancer cachexia progress [3]. In addition, IL-6 has an effect on the balance of energy and glucose [28] and can turn white adipose tissue into brown adipose tissue by driving uncoupling protein 1 [29]. We found that GE5, GE50 and Rb1 significantly reduced the levels of IL-6 (Fig. 6d, *P* < 0.01). GE5, GE50 and Rb1 are conducive to relieving the cancer cachexia symptom of systemic inflammation. The reduction of the two inflammatory cytokines indicates that the cancer cachexia state of tumor-bearing mice may have been improved by GE5, GE50 and Rb1.

A decrease in body weight and the skeletal muscle weight are the main symptoms of patients with cancer cachexia and are also the symptoms observed in the experimental C26 mouse model of cancer cachexia [30]. The reduction in the tumor-free body weight, epididymal fat and gastrocnemius muscles indicated that the model of cancer cachexia mice was successfully established by injecting C26 mouse colon cancer cells into BALB/c mice. WEG, GE5, GE50 and Rb1 did not improve the tumor-free weight of the mice (Fig. 1), and GE5, GE50 and Rb1 did not improve the weight of the gastrocnemius muscle and epididymal fat in cancer cachexia mice (Fig. 2). In addition, WEG, GE5, GE50 and Rb1 had no significant effect on food intake or tumor volume (Figs. 3 and 4). The results of the food intake study also indicated that the dietary status of the cancer cachexia mouse model did not significantly change during the period of cancer cachexia (Fig. 3). There was no significant increase in the diet, muscles or epididymal fat of the treatment group compared with those of the model group, and as a consequence, the tumor-free body weight of the treatment group mice did not increase significantly. Although no effect was found on improving the body weight of mice, we did not find a significant decrease in the body weight. We used ginseng extracts in other experimental animal models and found that ginseng can increase the body weight (data not yet published); thus, ginseng has the ability to increase the body weight of mice, but it did not give good results in this model. Although the tumors in the model group mice grew faster, the tumor-free weight of the model group mice was only significantly decreased compared to that of the control group on the 23rd day, indicating that the model has certain limitations. Because the symptoms of cancer cachexia mice are too severe to be improved with a short period of treatment, the treatment should be initiated as soon as possible, and the treatment duration should be longer.

This is one of the preliminary studies on the use of *Panax ginseng* (it has always been one of the most popular Chinese herbal medicines) to improve some of the adverse factors of cancer cachexia. In our study, to explore why prescriptions containing the Chinese traditional herbal medicine ginseng have an improved effect on cancer cachexia, we chose a water extract of ginseng, a ginsenoside mixture and a single ginsenoside for research. Because we found that GE5 and GE50 (Rg1: 3.49%, Re: 8.6%, Rf: 1.46%, Rb1: 14%, Rc: 16.4%, Rb2: 11.69%, and Rd.: 9.5%) have an improved effect on two inflammatory cytokines in a cancer cachexia mouse model, it is important to determine which compound played the major role. Although ginsenoside Rc has the highest content in GE5 and GE50, cancer-related research on this compound has rarely been conducted; the second highest compound was ginsenoside Rb1, and this was found to be associated with cancer. This is why we chose ginsenoside Rb1 to study whether it is the active ingredient. In addition, 10.72 mg/kg ginsenoside Rb1 is equivalent to the content of Rb1 in GE50, and this dose is also similar to the dose used in the ginsenoside Rb1-related literature [31, 32].

## Conclusions

In conclusion, we demonstrate that GE5 and GE50 can be used to reduce the levels of the TNF-α and IL-6 cytokines in cancer cachexia mice, and ginsenoside Rb1 can have the same effect; this may potentially be helpful in treating cancer cachexia. This study could also help us explore new herbal medicines for cancer cachexia therapies in the future.

## Supplementary information

**Additional file 1.** Quality control of water extract of ginseng (WEG).

**Additional file 2.** Certificate of Analysis.

## Data Availability

The datasets used and analyzed during the current study are available from the corresponding author on reasonable request.

## Abbreviations

DMEM: Dulbecco’s modified eagle’s medium
ELISAs: Enzyme-linked immunosorbent assays
GE5: Ginsenosides at doses of 5 mg/kg
GE50: Ginsenosides at doses of 50 mg/kg
IFN-γ: Interferon γ
IL-1: Interleukin 1
IL-6: Interleukin-6
PBS: Phosphate-buffered saline
Rb1: Ginsenoside Rb1
TNF-α: Tumor necrosis factor-α
WEG: Water extract of ginseng

## References

[1] Haehling SV, Anker SD (2014). Prevalence, incidence and clinical impact of cachexia: facts and numbers—update 2014. J Cachexia Sarcopenia Muscle.

[2] Kenneth F, Florian S, Anker SD, Ingvar B, Eduardo B, Fainsinger RL (2011). Definition and classification of cancer cachexia: an international consensus. Lancet Oncol.

[3] Pinto NI, Carnier J, Oyama LM, Otoch JP, Alcântara PS, Tokeshi F (2015). Cancer as a proinflammatory environment: metastasis and cachexia. Mediat Inflamm.

[4] Cong M-H, Zou B-H, Yu L (2016). Mechanisms of anorexia cancer cachexia syndrome and potential benefits of traditional medicine and natural herbs. Curr Pharm Biotechnol.

[5] Oluwadamilola O, White JD (2011). Herbal therapy use by cancer patients: a literature review on case reports. Eur J Cancer.

[6] Ernst E, Cassileth B (2015). The prevalence of complementary/alternative medicine in cancer: a systematic review. Cancer.

[7] Wang H, Chan YL, Li TL, Wu CJ (2012). Improving cachectic symptoms and immune strength of tumour-bearing mice in chemotherapy by a combination of Scutellaria baicalensis and Qing-Shu-Yi-Qi-Tang. Eur J Cancer.

[8] Zhang YN, Han XC, Ouyang B, Wu Z, Yu H, Wang Y (2017). Chinese herbal medicine Baoyuan Jiedu Decoction inhibited muscle atrophy of cancer cachexia through Atrogin-l and MuRF-1. Evid Based Complement Alternat Med.

[9] Terawaki K, Kashiwase Y, Sawada Y, Hashimoto H, Yoshimura M, Ohbuchi K (2017). Development of ghrelin resistance in a cancer cachexia rat model using human gastric cancer-derived 85As2 cells and the palliative effects of the Kampo medicine rikkunshito on the model. PLoS One.

[10] Ohbuchi K, Nishiumi S, Fujitsuka N, Hattori T, Yamamoto M, Inui A (2015). Rikkunshito ameliorates cancer cachexia partly through elevation of glucarate in plasma. Evid Based Complement Alternat Med.

[11] Choi YK, Jung KY, Woo SM, Yun YJ, Jun CY, Park JH (2014). Effect of Sipjeondaebo-tang on cancer-induced anorexia and cachexia in CT-26 tumor-bearing mice. Mediat Inflamm.

[12] Cheng K-C, Li Y-X, Cheng J-T (2012). The use of herbal medicine in cancer-related anorexia/ cachexia treatment around the world. Curr Pharm Des.

[13] Qi F, Li A, Inagaki Y, Gao J, Li J, Kokudo N (2010). Chinese herbal medicines as adjuvant treatment during chemo- or radio-therapy for cancer. Biosci Trends.

[14] Hofseth LJ, Wargovich MJ (2007). Inflammation, cancer, and targets of ginseng. J Nutr.

[15] Yao FD, Yang JQ, Huang YC, Luo MP, Yang WJ, Zhang B (2019). Antinociceptive effects of ginsenoside Rb1 in a rat model of cancer-induced bone pain. Exp Ther Med.

[16] Chen SF, Li X, Wang YL, Mu PW, Chen CJ, Huang PJ (2019). Ginsenoside Rb1 attenuates intestinal ischemia/reperfusion-induced inflammation and oxidative stress via activation of the PI3K/Akt/Nrf2 signaling pathway. Mol Med Rep.

[17] Wu YZ, Yu YH, Szabo A, Han M, Huang XF (2014). Central inflammation and leptin resistance are attenuated by ginsenoside Rb1 treatment in obese mice fed a high-fat diet. PLoS One.

[18] Tan SJ, Yu WK, Lin ZL, Chen QY, Shi JL, Dong Y (2014). Anti-inflammatory effect of ginsenoside Rb1 contributes to the recovery of gastrointestinal motility in the rat model of postoperative ileus. Biol Pharm Bull.

[19] Friedl R, Moeslinger T, Kopp B, Spieckermann PG (2010). Stimulation of nitric oxide synthesis by the aqueous extract of Panax ginseng root in RAW 264.7 cells. Br J Pharmacol.

[20] Fearon KCH (2012). The 2011 ESPEN Arvid Wretlind lecture: cancer cachexia: the potential impact of translational research on patient-focused outcomes. Clin Nutr.

[21] Tanaka Y, Eda H, Tanaka T, Udagawa T, Ishikawa T, Horii I (1990). Experimental cancer cachexia induced by transplantable colon 26 adenocarcinoma in mice. Cancer Res.

[22] Argilés JM, Stemmler B, López-Soriano FJ, Busquets S (2015). Nonmuscle tissues contribution to cancer cachexia. Mediat Inflamm.

[23] Inui A (1999). Cancer anorexia-cachexia syndrome: are neuropeptides the key?. Cancer Res.

[24] Chen C, Wang BB (2018). Brucea javanica oil emulsion alleviates cachexia induced by Lewis lung cancer cells in mice. J Drug Target.

[25] Li B, Wan LL, Li Y, Yu Q, Chen PG, Gan R (2014). Baicalin, a component of Scutellaria baicalensis, alleviates anorexia and inhibits skeletal muscle atrophy in experimental cancer cachexia. Tumor Biol.

[26] Baracos VE, Martin L, Korc M, Guttridge DC, Kch F (2018). Cancer-associated cachexia. Nat Rev Dis Primers.

[27] Patel HJ, Patel BM (2016). TNF-α and cancer cachexia: molecular insights and clinical implications. Life Sci.

[28] Abdullahi A, Jeschke MG (2016). White adipose tissue browning: a double-edged sword. Trends Endocrinol Metab.

[29] Maria T, Graham R (2013). Cancer cachexia: malignant inflammation, tumorkines, and metabolic mayhem. Trends Endocrinol Metab.

[30] Zhuang P, Zhang J, Wang Y, Zhang M, Song L, Lu Z (2016). Reversal of muscle atrophy by Zhimu and Huangbai herb pair via activation of IGF-1/Akt and autophagy signal in cancer cachexia. Support Care Cancer.

[31] Zhou P, Xie W, Luo Y, Lu S, Dai Z, Wang R (2018). Inhibitory effects of ginsenoside Rb1 on early atherosclerosis in ApoE−/− mice via inhibition of apoptosis and enhancing autophagy. Molecules.

[32] Chen T, Xiao L, Zhu L, Ma S, Yan T, Ji H (2015). Anti-asthmatic effects of ginsenoside Rb1 in a mouse model of allergic asthma through relegating Th1/Th2. Inflammation.

