# Supplementary material for: Ginsenoside Rb1 can ameliorate the key inflammatory cytokines TNF-α and IL-6 in a cancer cachexia mouse model
Source: BMC Complement Med Ther. 2020 Jan 15;20:11. doi: 10.1186/s12906-019-2797-9 (PMC7076885; doi:10.1186/s12906-019-2797-9)
Supplement: Supplementary file 1 — Additional file 1. Quality control of water extract of ginseng (WEG). [file 12906_2019_2797_MOESM1_ESM.docx]

Quality control of water extract of ginseng (WEG)

According to the method of the Pharmacopoeia of the People's Republic of China (2015), 1.0 mg / mL ginsenoside Re standard mother liquor was prepared using methanol as a solvent. The mother liquor was serially diluted with methanol to obtain a serial standard solution with a concentration of 0.02, 0.05, 0.1, 0.5, 1.0 mg/mL. The standard solution was sequentially entered into the HPLC system to obtain a chromatogram.

The standard curve equation is: y = 3433.4x-37565, R² = 0.9997, and the linear relationship is good in the range of 0.02-1.0 mg / mL, as shown in Figure.


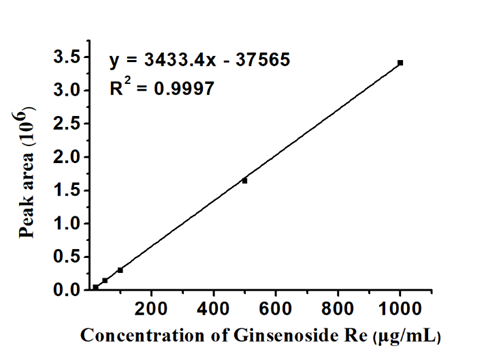


Six samples of water extract of ginseng (WEG) were prepared in parallel using the method (line-104-108). All samples were filtered through a 0.22 μm microporous membrane, followed by HPLC detection to obtain the chromatogram corresponding to each sample. The peak area value of each sample with a retention time of about 50.785 min was brought into y = 3433.4x-37565. After conversion, the respective ginsenoside Re concentrations were obtained, and the average and standard deviation were calculated.

The concentration of ginsenoside Re was 138.55 ± 8.64 μg/mL. It can be seen that the content of ginsenoside Re in the ginseng water extract prepared by the water extraction method is relatively stable, indicating that the method for preparing the ginseng water extract is feasible.
