# Supplementary material for: Ginsenoside Rb1 can ameliorate the key inflammatory cytokines TNF-α and IL-6 in a cancer cachexia mouse model
Source: BMC Complement Med Ther. 2020 Jan 15;20:11. doi: 10.1186/s12906-019-2797-9 (PMC7076885; doi:10.1186/s12906-019-2797-9)
Supplement: Supplementary file 2 — Additional file 2. Certificate of Analysis. [file 12906_2019_2797_MOESM2_ESM.doc]

Certificate of Analysis

| Product Name | Ginseng Extract |
| --- | --- |
| Batch Number | FY170314-B12 |
| Quantity | 60 kg |
| ManufactureDate | 14 March 2017 |
| Certificate Date | 03 May 2017 |

| Analysis | Specification | Results |
| --- | --- | --- |
| Assay (HPLC) | ≥65%  Rg1  Re  Rf  Rb1  Rc  Rb2  Rd | 65.32%  3.49%  8.6%  1.64%  14%  16.4%  11.69%  9.5% |
| Physical & Chemical Control |  | |
| Identification | Positive reaction | Verified |
| Appearance | Powder | Complies |
| Color | Canary yellow | Complies |
| Loss On Drying | ≤5% | Complies |
| Mesh Size | 80mesh | Complies |
| Heavy Metals | ≤10ppm | Complies |
| Microbiological |  | |
| TotalPlate Count | ≤1000cfu/g | Complies |
| Yeast & Mold | ≤100cfu/g | Complies |
| Salmonella | Negative | Negative |
| E.Coli | Negative | Negative |
| Shelf life | 36 Months |  |
| Storage | Store in a cool and dry place, keep away from strong light &heat | |


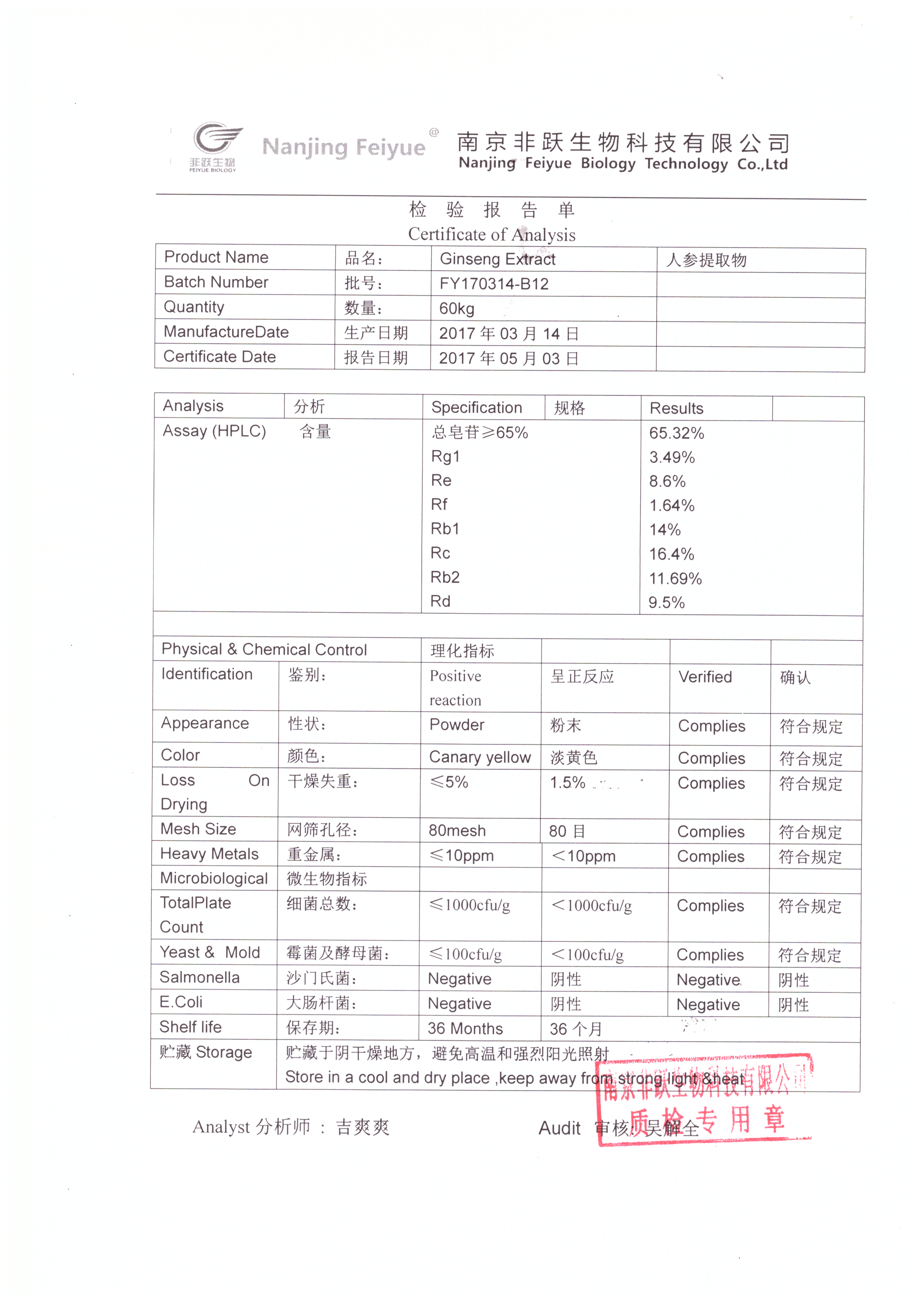
Certificate of Analysis-original
